# Effects of TGF-β1 and IGF-1 on proliferation of human nucleus pulposus cells in medium with different serum concentrations

**DOI:** 10.1186/1749-799X-1-9

**Published:** 2006-10-26

**Authors:** Rongfeng Zhang, Dike Ruan, Chao Zhang

**Affiliations:** 1 Department of Orthopaedic Surgery, Navy General Hospital of PLA, Beijing, 100037, People's Republic of China

## Abstract

**Background:**

The low proliferative viability of human nucleus pulposus(NP) cells is considered as a cause of intervertebral discs degeneration. Growth factors, such as TGF-β1 and IGF-1, have been implicated in cell proliferation and matrix synthesis.

**Objective:**

To investigate the dose-response and time-course effect of transforming growth factorβ1(TGF-β1) and insulin-like growth factor-1(IGF-1) on proliferation of NP cells.

**Study design:**

3-(4,5-dimethylthiazolyl)-2,5-diphenyl-tetrazolium bromide (MTT) is reduced by dehydrogenase in mitochondria of live cells. The proliferative viability of cells corresponds to the amount of MTT reduced, which is measured with an enzyme-linked immunosorbent assay plate reader. In this study, we assessed dose- and time-dependent effects of NP cells to TGF-β1 and IGF-1 in medium with different serum concentrations by MTT assay.

**Methods:**

After release of informed consent, tissue samples of NP were obtained from anterior surgical procedures performed on five donors with idiopathic scoliosis. Isolated cells were cultured in F12 medium supplemented with 10% fetal bovine serum(FBS). Cells were seeded in 96-well plates at 1 × 10^3 ^cells/well. After synchronization, medium was replaced by F12 containing 1% or 10% FBS with either single or combination of TGF-β1 and IGF-1. Dose-response and time-course effect were examined by MTT assay.

**Results:**

In the presence of 1% FBS, the response to IGF-1 was less striking, whereas TGF-β1 had a remarkably stimulating effect on cell proliferation. In 10% FBS, both of the two growth factors had statistical significant mitogenic effects, especially TGF-β1. The dose-dependent effect of TGF and IGF on cell proliferation was found within different concentrations of each growth factor(TGF-β1 1–10 μg/L, IGF-1 10–100 μg/L). The time-course effect showed a significant elevation three days later.

**Conclusion:**

TGF-β1 and IGF-1 were efficient to stimulate cell proliferation of human NP cells in vitro with a dose- and time-dependent manner. These results support the therapeutic potentials of the two growth factors in the treatment of disc degeneration.

## Background

Intervertebral disc(IVD) degeneration and associated spinal disorders are a leading source of morbidity, resulting in substantial pain and increased health care costs. The exact mechanism of disc degeneration is not fully understood. The NP tissue is avascular, gelatinous and lies in the central of the IVD. Like the articular cartilage, NP receives all nutrients by diffusion in bulk flow patterns[1]. As a result, NP tissue is prone to degenerate. A central feature of NP degeneration is loss of tissue cellularity. It has been suggested that apoptosis may be an important event that contributes to the death of cells in the disc[2].

Growth factors, such as TGF-β1 and IGF-1, have been shown to stimulate chondrocytes and endotheliocytes proliferation and matrix synthesis in vitro[3-5]. Many researchers have studied the effects of growth factors on NP cells, but a large number of them were confined to the effect of single factor on cell phenotype, furthermore, the exprimential objects were mainly rodent animals [6,7]. To investigate the therapeutic potential in the treatment of disc degeneration, we investigated the effects of TGF-β1 and IGF-1 on the proliferation of human NP cells in single or combination by MTT colorimetric assay. The assay detects the reduction of MTT by mitochondrial dehydrogenase to blue formazan product, which reflects the normal functioning of mitochondria and hence cell proliferation[8]. Different culture conditions were used to assess the influence of changes in the external environment of the cells on their responsiveness to growth factors. Growth factors were added in increasing concentrations to the culture medium to study their dose-response and time-course effect on NP cells.

## Materials and methods

### Cell isolation and culture

Protocol for the experimental study was approved by our institutional Research Review Committee. IVD specimens were obtained from anterior surgical procedures performed on five donors with idiopathic scoliosis(3 females and 2 males; average age, 15.4 years; range, 11–19 years). Specimens were transported in a sterile tube to the laboratory less than 30 min after surgical removed. The annulus fibrosus and transition zone was removed with scalpel. The NP tissue was careful separated from upper and lower vertebral cartilage under a binocular microscope. After rinsed twice in Ham's/F12(Hyclone) to remove residual debris, NP tissue was minced with a scalpel into small portions(1–2 mm2) and digested for 30 minutes at 37°C in 0.25% trypase(Gibco), followed by 4 hours in 0.2% collagenase Type II (Gibco). The digest was filtered through a 75-**μ**m cell-strainer and cultured in T25 flasks(Costar) at a density of 1 × 104 cells/ml in F12 containing 10% FBS(Hyclone) in a 37°C, 5%CO2 atmosphere. The medium was changed every 72 hours. When cultures showed a 80% confluency, cells were trypsinized and a split ratio of 1:4 was used for subculturing. Cell viability, determined by trypan blue(Hyclone) exclusion, averaged 96% on monolayer culture. Cellular morphology cultured in 96-well plates was observed microscopically every day so as to observe the effects of growth factors on cells.

### Effects of TGF-β1 and IGF-I on cell proliferation

After reaching 80% confluency, the cells(passage = 2) were trypsinized and cultured in 96-well plates at a density of 1 × 10^4^/ml in F12 containing 10% FBS with 0.1 ml per well. After 24 hours of adherence, the medium was changed by F12 without FBS and the cells were cultured for another 12 hours to ensure the cells synchronization.

#### 1. Dose-response effect of growth factors on cell proliferation

A 96-well plate with synchronal NP cells was taken and the medium was removed, different concentrations of growth factors dispensed by F12 with 1% or 10% FBS were added to the plate. The TGF-β1 (Peprotech) and IGF-1 (Peprotech) groups were all divided into eight concentration groups: 0.1, 0.5, 1.0, 5.0, 10, 50, 100, 500 μg/L. F12 contained 1% or 10% FBS without growth factors was used as control groups. Every specimen was prepared in four replicates. After incubating cells for 72 hours, 20 μL of 5 mg/mL MTT (Sigma)in phosphate buffered saline was added to each well and the plate was incubated at 37°C for 4 hours. The medium was removed and 100 μl of dimethyl sulfoxide(DMSO, Sigma) per dish was then added. After agitation at 25°C for 10 minutes, optical density in control and growth factor groups was assayed at 490 nm with an enzyme-linked immunosorbent assay plate reader (bio-tek instruments).

#### 2. Time-course effect of growth factors in single or combinations on cell proliferation

Four 96-well plates with synchronal cells were used. The medium was replaced by 10%FBS-F12 medium with growth factors in optimal effect-concentration(acquired by dose-effect experiment). This experiment was divided into 4 groups: control group, 10 μg/L TGF-β1, 100 μg/L IGF-1, 10 μg/L TGF-β1+ 100 μg/L IGF-1. Each condition was repeated 4 times. The plates were incubated at 37°C. Assay was carried out at 1-, 3-.5- and 7-day using above mentioned methods.

### Statistical analyses

Standard statistical analytical methods were performed using the SPSS11.0 system for windows. The effects of different groups were assessed with a one-way analysis of variance(ANOVA) followed by a Fisher LSD *post hoc *test. Signifcance was assumed when p < 0.05.

## Results

### Dose-response effect of growth factors on cell proliferation

**Figure 1:**
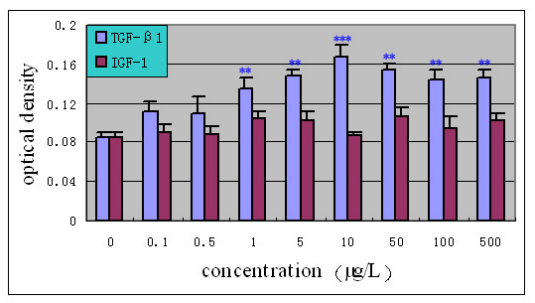
**Dose-response effect of growth factors on proliferation of human NP cells (1%FBS/F12) (n = 20)**. Cells were incubated with increasing concentrations (0.1–500 μg/L) of TGF-β1 and IGF-I in 1%FBS/F12 medium for 72 hours. The proliferative response was evaluated using MTT assay. Results were expressed as the mean optical density of MTT absorbance in growth factors and control wells. ** *P *< 0.01, *** *P *< 0.001 versus control.

Under 1% FBS condition, the optical density of the control group was 0.085 with a lower level of viability, as shown in Fig. 1. Notably, there was no statistical significance between the IGF-1 group and the control group. The 10 μg/L TGF-β1 group showed marked elevation in proliferation compared to the control group (96.5%).

**Figure 2:**
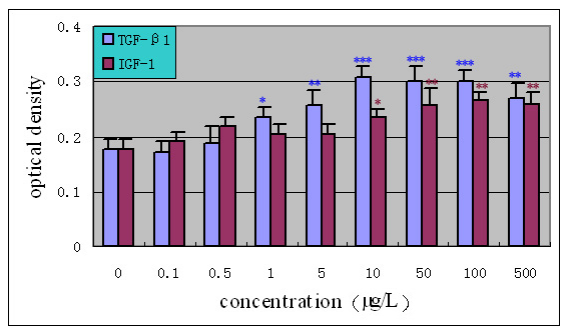
**Dose-response effect of growth factors on proliferation of human NP cells (10%FBS/F12) (n = 20)**. Cells were incubated with increasing concentrations (0.1–500 μg/L) of TGF-β1 and IGF-I in 10%FBS/F12 medium for 72 hours. The proliferative response was evaluated by MTT assay. Results were expressed as the mean optical density of MTT absorbance in growth factors and control wells. * *P *< 0.05, ** *P *< 0.01, *** *P *< 0.001 versus control.

In the presence of 10% FBS, the optical density of the control group had an obvious elevation and reached 0.178, about twice the optical density in the 1% FBS. The viability of the cells had a further elevation as a result of exposing to growth factors. As shown in Fig. 2, a significant stimulating effect of TGF-β1 over doses of 1.0–500.0 μg/L on NP cells, and the maximum stimulus with 10 μg/L of the TGF-β1 reached a 72.4% increase compared with the standard medium. The results also showed a mitogenic effect of IGF-1 over doses of 10.0–500 μg/L after 72 hours exposure. Cells showed the most marked elevation in proliferation at the concentration of 100 μg/L IGF-1 compared to controls(50.6%). Dose-dependent effects of TGF-β1 and IGF-1 on cell proliferation were found between 1–10 μg/L and 10–100 μg/L respectively. However, cell viability showed a trend of decreasing in response to further elevation of the concentrations.

### Time-course effect of growth factors in single or combinations

**Figure 3:**
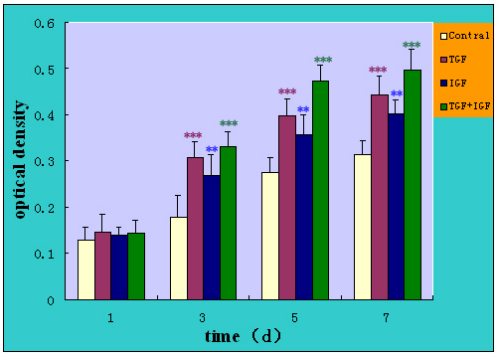
**Time-course effect of growth factors on proliferation of human NP cells (10%FBS/F12) (n = 20)**. The proliferative response was evaluated by MTT assay at 1-, 3-.5- and 7-day. Results were expressed as the mean optical density of MTT absorbance in growth factors in single or combinations and control wells. * *P *< 0.05, ** *P *< 0.01, *** *P *< 0.001 versus control.

The optical density was assessed when NP cells were cultured in medium supplemented with growth factors in single or combinations in their optimal concentrations (10 μg/L TGF-β, 100 μg/L IGF-1). Cell proliferation didn't show significent increase at the first day of culture when either growth factor was added to the medium(as shown in Fig. 3). In the medium with 10% FBS, the responsiveness of NP cells to the growth factors showed a significant increase compared to the control group after 3, 5, and 7 days of culture. Compared with the data of standard medium at the 3 days, the proliferative percence of TGF-β1, IGF-1 and TGF-β1+IGF-1 on cell proliferation was calculated as 72.4% (P < 0.001), 50.6% (P < 0.01), and 86% (P < 0.001) respectively. The total amount of cell proliferation by growth factors also had a marked elevation at 5 and 7 days. The clear time-dependent increase with growth factors in the 10% FBS was observed. Stimulation by TGF-β1+IGF-1 group was the maximum respectiveness at every time point among growth factors, which reached 71.3% (P = 0.001) and 59.3 (P < 0.01) increase at 5 and 7 days compared to the standard medium.

### Effects of growth factors on cell morphology

**Figure 4:**
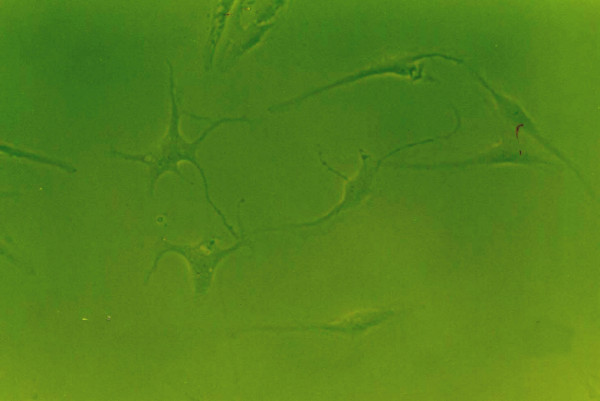
**The morphology of human NP cells under 1%FBS conditions**. 1%FBS, NP cells lost their normal morphology and trended to irregular.(×100).

**Figure 5:**
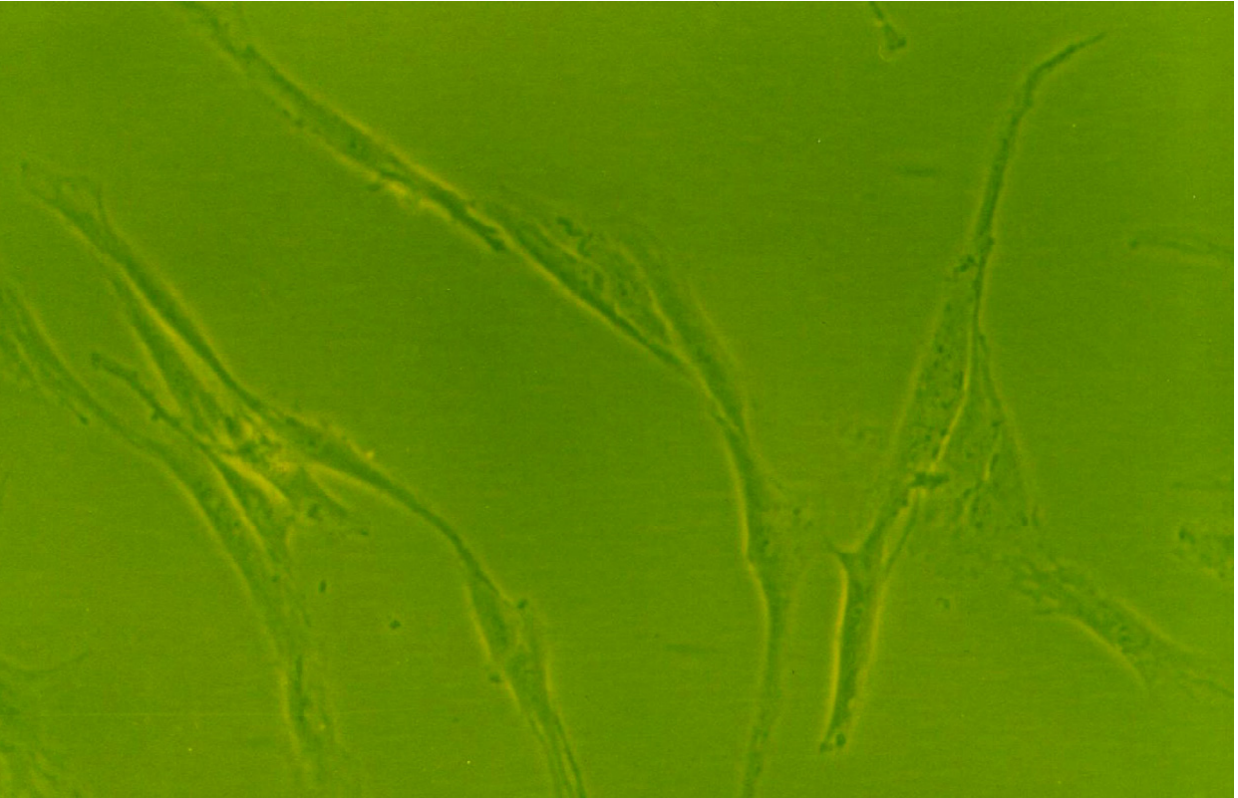
**The morphology of human NP cells under 1%FBS conditions**. 1%FBS, 10 μg/L TGF-β1, NP cells were similar to the normal NP cells with regular spindle-shape. (×100).

**Figure 6:**
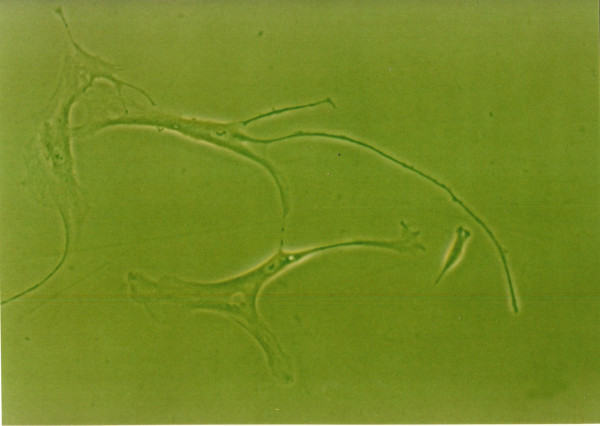
**The morphology of human NP cells under 1%FBS conditions**. 1%FBS, 100 μg/L IGF-1, NP cells showed an irregular morphology with low mitotic activity. (×100).

**Figure 7:**
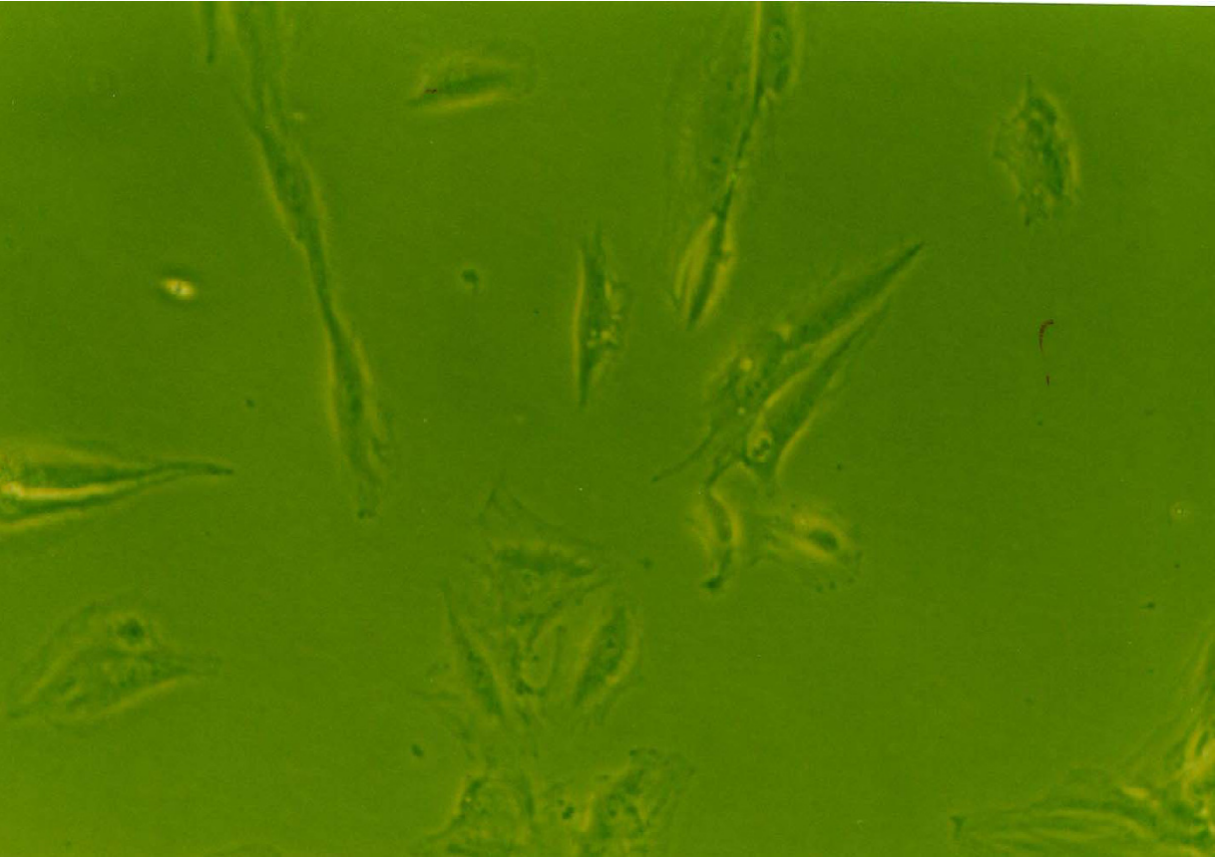
**The morphology of human NP cells under 1%FBS conditions**. 1%FBS, 10 μg/L TGF-β1+100 μg/L IGF-1, NP cells trended to appear in short spindle-shape with short pseudopods. (×100).

**Figure 8:**
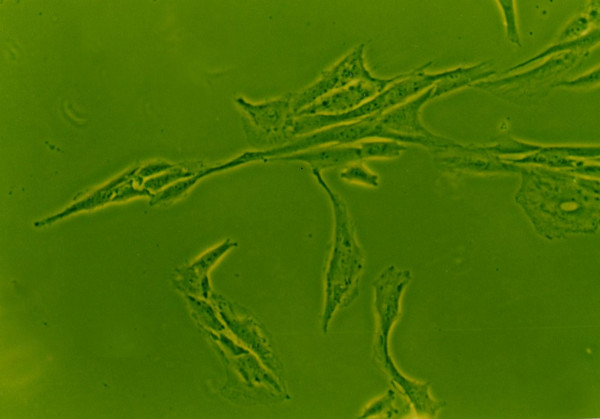
**The morphology of human NP cells under 10%FBS conditions**. 10%FBS, NP cells were spindle-shaped with abundant cytoplasm and two pseudopods. (×100).

When grown in monolayer culture and standard medium, cells from normal human NP were spindle-shaped with abundant cytoplasm and two pseudopods(Fig. 8). Under 1% FBS conditions, the control group NP cells lost their normal morphology and trended to irregular, such as the obviously enlongate pseudopods and decreased cytoplasm(Fig. 4). The morphology of cells exposure to IGF-1 showed no striking difference compared with the control group(Fig. 6). However, cells in 1% FBS exposure to TGF-β1 or TGF-β1+IGF-1 were similar to the normal NP cells with regular spindle-shape (Fig. 5, 7).

**Figure 9:**
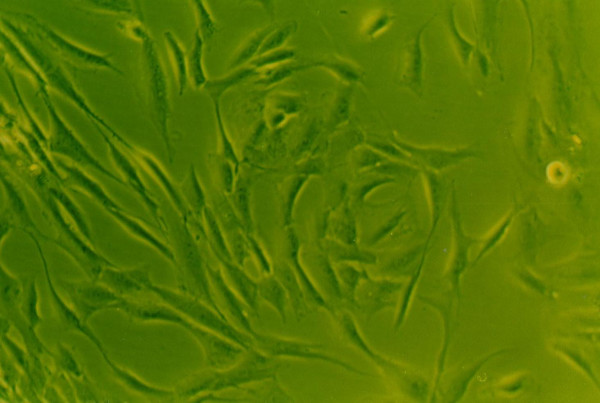
**The morphology of human NP cells under 10%FBS conditions**. 10%FBS, 10 μg/L TGF-β1, NP cells trended to appear in short spindle-shape or multiangular shape with short pseudopods and aboundant cytoplasm compared to the control group. (×100).

**Figure 10:**
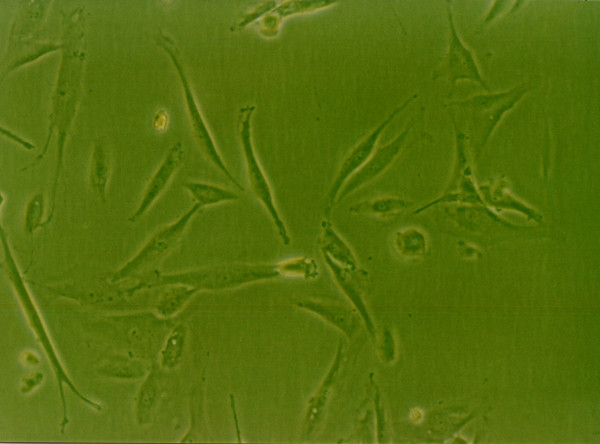
**The morphology of human NP cells under 10%FBS conditions**. 10%FBS, 100 μg/L IGF-1, the morphology of NP cells were similar to the 10 μg/L TGF-β1 group. (×100).

**Figure 11:**
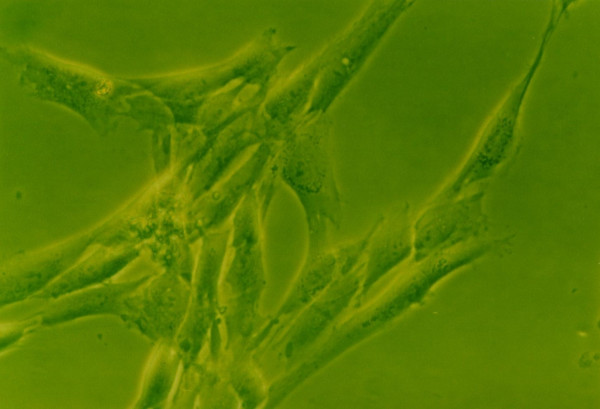
**The morphology of human NP cells under 10%FBS conditions**. 10%FBS, 10 μg/L TGF-β1+100 μg/L IGF-1, NP cells showed a maximum changes in morphology with short pseudopods and aboundant cytoplasm. (×200).

The morphology of cells exposure to growth factors had little change compared with the control group in the medium containing 10% FBS in the early culture stage(<72 hours). Whereas 72 hours later, the morphology of growth factors groups trended to appear in short spindle-shape or multiangular shape with pseudopods shorter, cytoplasm aboundant and refractive power stronger compared to the control group(Fig. 9, 10). Cells of exposure to TGF-β1(10 μg/L) + IGF(100 μg/L) showed a maximum changes in morphology(Fig. 11). No big difference was noted in cell morphology between TGF-β1 and IGF-1 groups.

## Discussion

In the present study, we observed profound changes in cell morphology and proliferation in samples cultured within medium containing TGF-β1. This is well supported also by previous studies in the literature. Joon et al[9] cultured human IVD cells in monolayer, alginate beads, or pellets, found proteoglycan synthesis increased 150–180%, 250–315%, or 375–425% over their controls respectively at 3 weeks. The pellets also increased synthesis of collagen Type II in response to TGF-β1. More recently, Tan et al[10] found a significant increase of the proteoglycan and collagen synthesis after infection of Ad/CMV-hTGF encoding gene into the degenerated disc cells, the structure and function of the degenerated cells changed remarkably. Alini et al[11] observed an increase and maintain in density of bovine NP cells and synthesis of proteoglycan. Gu[12] reported a stimulatory effect in proteoglycan core protein gene expression of TGF-β1 on the high-passage dedifferential NP cells. In addition, the dose-dependent effect of TGF-β1 on cell proliferation was found between the concentration ranges of 1.0–10 μg/L, This range is very important in the future therapeutic applications of the TGF-β1 in human disc degeneration, and has not been reported previously.

It has long been appreciated that NP tissue is a low nutrition region. In this in vitro study, when NP cells was cultured in medium with 1% FBS(necessary for basal cell maintenance, the cells showed a morphologic characteristics of nutrition deficiency. However, adding TGF(10 μg/L) in the medium resulted in a total recovery of cell morphology to that of normal cells cultured in standard medium and a 96.5% increase of cell proliferation over the control group. The results suggested that TGF-β1 play an important role in mitogensis at low nutrient conditions.

IGF-1 has been reported to stimulate cell proliferation and matrix synthesis, maintain cell phenotype in vitro[13]. Osada et al[14] demonstrated the generation and expression of IGF-1 mRNA in the bovine NP cells. The study showed an autocrine/paracrine mechanism of IGF-1 secretion, and the stimulative effect of IGF-1 on proteoglycan synthesis in bovine IVD. More recently, Gruber[15] demonstrated a significant reduction in the percentage of apoptotic disc cells after exposure to 50–500 ng/ml IGF-1 and suggested that IGF-1 could retard or prevent programmed cell death in vitro. The results presented in this report showed the response to IGF-1 was less striking in 1%FBS, but a marked elevation in cell proliferation and the dose-dependent effect was found between the concentrations of 10–100 μg/L IGF-1 in the presence of 10% FBS. However, cell viability decreased with further raise of the concentration. This probably related to the bound state of cell surface receptors. No significant effect in 1% FBS revealed that the proliferative effect of IGF-1 perhaps rely on other components in the serum.

Irrespective of which growth factor was added, no significant stimulation of this proliferation rate was observed at the first day of our experiments. This may be a delayed response of growth factors on cell proliferation. The exact mechanism is not fully understood at present.

The final goal of the present study is to provide the basis for the development of an efficient application of growth factors in the treatment of IVD degeneration. Firstly, the use of growth factors by intradiscal application in vivo might help the disc increase cell proliferation and stimulate matrix production, especially for individuals in the early stage of degeneration found by MRI examination[16]. An[17] reported that the intradiscal administration of osteogenic protein-1 by injection in vivo resulted in an increased disc height present at 2, 4, and 8 weeks and an increase in proteoglycan content of the rabbit nucleus pulposus at the 2-week time point. This demonstrated the potential in vivo effects of growth factors on the IVD degeneration by direct injection approach. Secondly, tissue-engineering and gene therapy techniques could offer a sustained release of growth factors, and the results from the present study could be used in the regeneration of the lost functional capacity of the degenerative IVD. However, seed cells continue to be the problems, such as limited source, minor amounts and lower viability. According to this study, TGF-β1 and IGF-1 are effective factors to stimulate proliferation of the seed cells.

In summary, the results demonstrate the effectiveness of TGF-β1 and IGF-1 in stimulating cell proliferation and maintaining morphology of human NP cells. There exist an optimal range for cell stimulation, and a synergic effect was observed when two growth factors were applied at the same time. These results provide useful information for possible clinical application in future. However, further studies such as the effects of growth factors on degenerative disc tissue in vivo, and the delivery carrier of the growth factors should be performed in small and large animals.

## Abbreviations

MTT = methyl thiazolyl tetrazolium; TGF-β1 = transforming growth factorβ1; IGF-1 = insulin-like growth factor-1; NP = nucleus pulposus; FBS = fetal bovine serum; IVD = Intervertebral disc.

## Competing interests

national natural science funds were received in support of this work. No benefits in any form have been or will be received from a commercial party related directly or indirectly to the subject of this report.

## Authors' contributions

R.F. Zhang carried out the cell culture and MTT assay, participated in the acquisition of data and drafted the manuscript. Z. Chao participated in the design of the study and performed the statistical analysis. D.K. Ruan conceived of the study, participated in its design and revised the manuscript. All authors read and approved the final manuscript.
